# Reproductive Shifts and Ovarian Cancer Risk in Women Aged 40 Years or Older

**DOI:** 10.1001/jamanetworkopen.2025.56840

**Published:** 2026-02-03

**Authors:** Jin-Hwi Kim, In-Sun Hwang, Su-Jeong Lee, Chan-Joo Kim, Sung-Jong Lee, Kyungdo Han

**Affiliations:** 1 Division of Gynecologic Oncology, Department of Obstetrics and Gynecology, Uijeongbu St Mary’s Hospital, College of Medicine, The Catholic University of Korea, Seoul, Korea; 2 Division of Gynecologic Oncology, Department of Obstetrics and Gynecology, Seoul St Mary’s Hospital, College of Medicine, The Catholic University of Korea, Seoul, Korea; 3 Department of Statistics and Actuarial Science, Soongsil University, Seoul, Korea

## Abstract

**Importance:**

Reproductive factors are associated with ovarian cancer risk, but their influence may differ across menopausal status and birth cohorts.

**Objective:**

To examine the associations between reproductive factors and ovarian cancer risk stratified by menopausal status and birth cohort.

**Design, Setting, and Participants:**

This nationwide population-based cohort study obtained data from the National Health Insurance Service (NHIS), a single-payer system covering 97% of the population in South Korea. Women aged 40 years or older who underwent NHIS health screening in 2009 and had reproductive, clinical, and other data were included and followed up until ovarian cancer diagnosis, death, or December 31, 2022. Data were analyzed in March 2025.

**Exposures:**

Age at menarche, parity, breastfeeding duration, oral contraceptive use, age at menopause, total reproductive span, and hormone replacement therapy use.

**Main Outcomes and Measures:**

Incident ovarian cancer identified from NHIS claims with *International Statistical Classification of Diseases and Related Health Problems, Tenth Revision* codes C56, C57, and C48 and confirmed through the Rare/Intractable Disease Registry (code V193). Cox proportional hazards regression models estimated hazard ratios (HRs) and 95% CIs.

**Results:**

A total of 2 285 774 women (932 637 [40.8%] premenopausal, 1 353 137 [59.2%] postmenopausal; mean [SD] age, 54.9 [10.85] years) were included in the final analytic cohort. The mean (SD) follow-up duration overall was 10.7 (2.99) years, and 10 729 ovarian cancer cases were identified during follow-up. Early menarche (aged ≤12 vs >16 years) was associated with higher ovarian cancer risk in both premenopausal women (HR, 1.37; 95% CI, 1.16-1.61) and postmenopausal women (HR, 1.24; 95% CI, 1.00-1.54). Parity of 2 or more births was associated with lower risk in both groups (HR, 0.68 [95% CI, 0.58-0.79] and 0.71 [95% CI, 0.60-0.85]). Breastfeeding for 12 months or longer and oral contraceptive use for 1 year or longer were associated with lower risk in premenopausal women but not postmenopausal women (HR, 0.86 [95% CI, 0.77-0.96] and 0.75 [95% CI, 0.61-0.93]). Among postmenopausal women, later menopause (at age ≥55 years; HR, 1.36 [95% CI, 1.11-1.66]), longer reproductive span (≥40 years; HR, 1.21 [95% CI, 1.09-1.34]), and hormone replacement therapy use for 2 to 5 years (HR, 1.20 [95% CI, 1.07-1.34]) were associated with higher risk. Parity-related risk reduction was attenuated in the 1960s birth cohort (HR, 1.07; 95% CI, 0.52-2.19; *P* for interaction = .36).

**Conclusions and Relevance:**

This cohort study found that reproductive factors were associated with ovarian cancer risk, with distinct patterns across menopausal status and birth cohorts. These findings highlight the need for tailored prevention strategies in aging, low-fertility populations.

## Key Points

**Question:**

What are the associations between reproductive factors and ovarian cancer risk across menopausal status and birth cohorts?

**Findings:**

In this cohort study of 2 285 774 women in Korea aged 40 years or older, early menarche, later menopause, and longer reproductive span were associated with higher ovarian cancer risk, whereas parity of 2 or more births was associated with lower risk across menopausal status groups. Breastfeeding duration and oral contraceptive use were associated with lower risk only in premenopausal women, and the inverse association for parity was attenuated in the 1960s cohort.

**Meaning:**

The findings of this study support the need for tailored prevention strategies in aging, low-fertility societies.

## Teaser

This cohort study identifies the reproductive factors associated with ovarian cancer risk among premenopausal and postmenopausal women born in different generations in South Korea.

## Introduction

Ovarian cancer remains one of the most lethal gynecologic malignant neoplasms worldwide, and incidence continues to increase in several East Asian countries despite declining rates in many high-income settings.^1,2,3,4^ Reproductive factors—including age at menarche, parity, breastfeeding duration, oral contraceptive (OC) use, age at menopause, total reproductive span, and hormone replacement therapy (HRT) use—have been consistently associated with ovarian cancer risk through mechanisms involving ovulatory activity, hormonal exposure, and inflammation-related pathways.^5,6,7,8,9^ Meta-analyses have reported a 5% to 8% higher risk per year with earlier menarche and a 20% to 30% lower risk with each additional birth, although most evidence derives from Western populations with historically higher fertility and greater OC use.^10,11^

Emerging demographic and epidemiologic data suggest that these associations may not be uniform across generations or menopausal phases. In low-fertility settings, fertility decline and delayed childbearing may shift lifetime ovulatory burden upward, while changes in breastfeeding practices and OC availability may alter exposure distributions across cohorts.^12,13,14^ Ecological data indicate a 30- to 40-year lag between fertility decline and increasing ovarian cancer incidence, raising questions about whether protective associations observed in older, high-parity cohorts persist in younger generations with substantially lower parity.^15,16,17^ Moreover, reproductive factors occurring early in life (eg, menarche, pregnancy, breastfeeding duration, and OC use) may play a greater role in cancer risk before menopause, whereas cumulative exposures (eg, reproductive span and HRT) may be more relevant after menopause.^10,18,19^

Korea provides a unique context for examining these patterns, having experienced one of the most rapid fertility declines globally—from a total fertility rate higher than 4.0 in the early 1970s to 0.72 in 2022—alongside increasing ovarian cancer incidence.^12,20,21^ Prior studies in Asian cohorts suggest that the inverse association for parity may be of lower magnitude in younger, low-fertility cohorts, but evidence remains limited and inconsistent.^11,17,18^ To our knowledge, no large-scale study has comprehensively evaluated whether reproductive factor associations differ by menopausal status and birth cohort within the same population.

We hypothesized that reproductive factors traditionally associated with ovarian cancer risk may exhibit distinct patterns across menopausal status and birth cohorts. In this study, using a nationwide cohort of Korean women with detailed reproductive histories and more than a decade of follow-up data, we aimed to assess the associations between reproductive factors and ovarian cancer risk to clarify generational differences and inform tailored prevention strategies for aging populations undergoing rapid demographic transition.

## Methods

The Catholic University of Korea, Uijeongbu St Mary’s Hospital Institutional Review Board approved this cohort study and waived the informed consent requirement because all data were anonymized. The study followed the Strengthening the Reporting of Observational Studies in Epidemiology (STROBE) reporting guideline.^22^

### Data Source and Study Population

This population-based cohort study obtained data from the Korean National Health Insurance Service (NHIS), a mandatory single-payer system covering approximately 97% of the South Korean population. The NHIS provides biennial health examinations for adults 40 years or older, including anthropometric measurements, self-administered questionnaires on lifestyle and reproductive history, and laboratory tests. The NHIS database includes eligibility information, claims with *International Statistical Classification of Diseases and Related Health Problems, Tenth Revision (ICD-10)* codes, health examination results, and mortality records.

Among women 40 years or older who underwent NHIS health screening in 2009, we excluded those with missing menopausal status, history of hysterectomy, outlier ages at menarche (<5 or ≥30 years) or menopause (<30 or >60 years), prior cancer diagnosis, missing key covariates from health examination or reproductive history, ovarian cancer diagnosis within 1 year of screening, or premenopausal status at age 55 years or older by the end of follow-up. The final analytic cohort included both premenopausal and postmenopausal women. Menopausal status (cessation of menstruation ≥1 year) was self-reported.

Follow-up began 1 year after the screening date (lag period) to reduce bias from undiagnosed cases and continued until ovarian cancer diagnosis, death, or December 31, 2022. Loss to follow-up was minimal (<0.5% annually), primarily due to emigration.

### Primary Outcome

The primary outcome was incident ovarian cancer. Cases were identified using NHIS claims with *ICD-10* codes C56, C57, and C48, encompassing ovarian, adnexal, and primary peritoneal malignant neoplasms, as commonly grouped in population-based studies. All cases were confirmed by enrollment in the national Rare/Intractable Disease Registry (code V193), a validated method for case ascertainment in Korean claims-based research. Participants were followed up from 1 year after the 2009 baseline screening—to minimize reverse causal association bias from subclinical disease—until ovarian cancer diagnosis, death, or December 31, 2022, whichever occurred first. The mean (SD) follow-up duration overall was 10.7 (2.99) years.

### Reproductive Factors

Reproductive factors were obtained from standardized self-administered questionnaires at the 2009 NHIS screening. Age at menarche, parity, breastfeeding duration, and OC use were categorized using standard NHIS response options. Among postmenopausal women, age at menopause, total reproductive span (age at menopause minus age at menarche), and HRT duration were similarly categorized.

### Covariates

Covariates were obtained from the 2009 NHIS health examination and included age, income level based on health insurance premiums (lowest quartile or medical-aid beneficiary), and place of residence (urban or rural). Lifestyle factors were assessed using standardized questionnaires and included smoking status (never, former, or current); alcohol consumption (none, mild intake of <20 g per day, or heavy intake of ≥20 g per day); and regular physical activity, which was defined as engaging in at least 30 minutes of moderate-intensity exercise on 5 or more days per week or at least 20 minutes of vigorous-intensity exercise on 3 or more days per week. Anthropometric and laboratory parameters measured by trained personnel after an overnight fast included height, weight, waist circumference, systolic and diastolic blood pressure, fasting glucose, total cholesterol, and serum creatinine. Body mass index (BMI) was calculated as weight in kilograms divided by height in meters squared. Comorbidities were identified using a combination of NHIS examination results and claims data: diabetes was defined as *ICD-10* codes E11 to E14, concomitant antidiabetic medication use, or fasting glucose of 126 mg/dL or greater (to convert to micromoles per liter, multiply by 0.0555); hypertension was defined as *ICD-10* codes I10 to I13 or I15, antihypertensive medication use, or blood pressure of 140/90 mm Hg or higher; dyslipidemia was defined as *ICD-10* code E78, lipid-lowering therapy, or total cholesterol of 240 mg/dL or higher (to convert to micromoles per liter, multiply by 0.0259); and chronic kidney disease was defined as an estimated glomerular filtration rate less than 60 mL/min/1.73 m^2^.

### Statistical Analysis

Baseline characteristics were summarized separately for premenopausal and postmenopausal women using means with SDs for continuous variables and numbers with percentages for categorical variables. Menopausal status was determined at baseline (2009). Because follow-up reproductive data are not updated in the NHIS, women who were premenopausal at baseline but reached 55 years or older—an indicator of natural menopause—were evaluated in a sensitivity analysis to reduce potential misclassification. Multivariable Cox proportional hazards regression models were used to estimate hazard ratios (HRs) and 95% CIs for ovarian cancer risk according to reproductive factors. Time on study was used as the underlying timescale. The proportional hazards assumption was evaluated with Schoenfeld residuals, and no violations were identified.^23^ Models were adjusted sequentially: model 1 was unadjusted; model 2 was adjusted for age; model 3 was adjusted for age, income level, smoking status, alcohol consumption, physical activity, diabetes, hypertension, dyslipidemia, and chronic kidney disease; and model 4 was adjusted as model 3 plus adjustment for reproductive factors, including age at menarche, parity, breastfeeding duration, and OC use (for postmenopausal women, age at menopause and HRT use were added). A 1-year lag period was applied to minimize reverse causality associated with subclinical disease. Subgroup analyses stratified by birth cohort (1930s-1960s) assessed generational variation, with interaction terms evaluated using likelihood ratio tests. Interactions between reproductive factors (eg, age at menarche with parity, breastfeeding duration, or OC use) were examined in both the premenopausal and postmenopausal groups using multivariable-adjusted interaction models. Additionally, national fertility patterns and ovarian cancer incidence were summarized descriptively, applying an approximate 40-year epidemiologic lag to contextualize generational changes. A proportion of women had missing information in self-reported reproductive or clinical variables, which are optional components of the NHIS health-screening questionnaire. Participants with such missing values were excluded from the primary analytic cohort.

To assess the robustness of findings, we conducted sensitivity analyses that (1) excluded women who were 55 years or older during follow-up in the premenopausal group, (2) also adjusted for BMI, and (3) restricted analyses to complete cases. All sensitivity analyses yielded results consistent with the primary findings.

All statistical analyses were performed using SAS, version 9.4 (SAS Institute Inc). A 2-sided *P* < .05 was considered statistically significant. Data analysis was conducted in March 2025.

## Results

**Figure 1.**
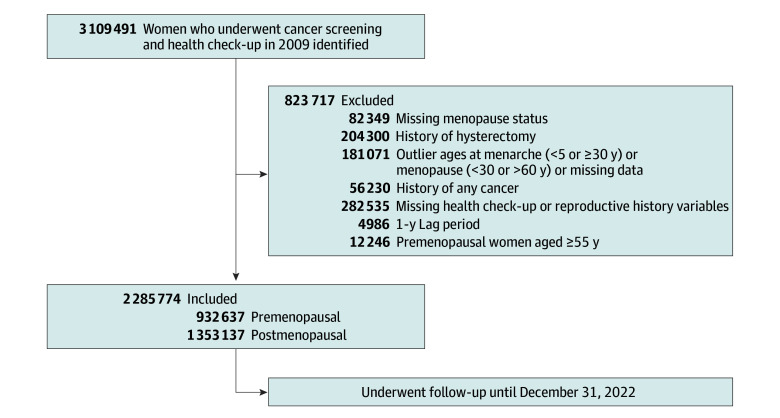
Study Flowchart

A total of 3 109 491 women aged 40 years or older participated in the 2009 NHIS health screening, of whom 823 717 were excluded from this study (Figure 1). The analytic cohort included 2 285 774 participants (mean [SD] age, 54.9 [10.85] years), including 932 637 (40.8%) in the premenopausal group and 1 353 137 (59.2%) in the postmenopausal group (Table 1). Premenopausal women were younger and predominantly born in the 1960s, whereas most postmenopausal women were born in the 1950s or earlier (mean [SD] age, 43.8 [2.96] vs 63.9 [2.87] years). Mean (SD) age at menarche was earlier in premenopausal women than in postmenopausal women (15.0 [1.62] vs 16.7 [1.82] years). Parity of 2 or more live births was reported by 82.9% of the premenopausal group and 92.4% of the postmenopausal group; nulliparity was more common among premenopausal participants. Breastfeeding duration of 12 months or longer was reported by 31.1% of premenopausal women and 70.8% of postmenopausal women. OC use of 1 year or longer remained uncommon in both groups but was more frequent among the premenopausal group (3.5% vs 6.3%). Postmenopausal women had higher BMI, greater central adiposity, more cardiometabolic comorbidities, and lower rates of alcohol consumption and current smoking (Table 1).

**Table 1.** Baseline Characteristics of Study Population

Characteristic	Women, No. (%)	
	Premenopausal	Postmenopausal
Total No.	932 637	1 353 137
Age, mean (SD), y	43.8 (2.96)	63.9 (2.87)
Birth cohort		
≤1930s	0	285 320 (21.1)
1940s	0	483 455 (35.7)
1950s	140 913 (15.1)	538 196 (39.8)
1960s	791 724 (84.9)	46 166 (3.4)
Income level: lowest quartile or medical-aid beneficiary	231 406 (24.8)	290 925 (21.5)
Urban place of residence	432 698 (46.4)	573 285 (42.4)
BMI, mean (SD)	23.1 (3.06)	24.2 (3.14)
Waist circumference, mean (SD), cm	77.8 (8.2)	80.6 (8.4)
Fasting glucose, mean (SD), mg/dL	92.4 (10.8)	95.2 (11.4)
SBP, mean (SD), mm Hg	119.5 (14.8)	126.3 (16.2)
DBP, mean (SD), mm Hg	73.2 (9.6)	76.8 (10.2)
Total cholesterol, mean (SD), mg/dL	198.5 (36.7)	210.2 (38.1)
Smoking status		
Never	885 485 (94.9)	1 302 083 (96.2)
Former	15 002 (1.6)	14 290 (1.1)
Current	32 150 (3.4)	36 764 (2.7)
Alcohol consumption		
None	666 951 (71.5)	1 186 775 (87.7)
Mild: <20 g/d	254 807 (27.3)	159 501 (11.8)
Heavy: ≥20 g/d	10 879 (1.2)	6861 (0.5)
Regular exercise	160 283 (17.2)	244 486 (18.1)
Comorbidities		
Diabetes	32 526 (3.5)	176 597 (13.1)
Hypertension	128 706 (13.8)	627 253 (46.4)
Dyslipidemia	99 059 (10.6)	444 739 (32.9)
Chronic kidney disease	26 552 (2.8)	160 965 (11.9)
Age at menarche, mean (SD), y	15.0 (1.62)	16.7 (1.82)
Parity		
0	35 156 (3.77)	22 800 (1.68)
1	124 035 (13.3)	79 986 (5.9)
≥2	773 446 (82.9)	1 250 351 (92.4)
Breastfeeding duration, mo		
None	170 932 (18.3)	86 200 (6.4)
<6	226 618 (24.3)	83 141 (6.1)
6 to <12	245 044 (26.3)	226 227 (16.7)
≥12	290 043 (31.1)	957 569 (70.8)
OC use, y		
None	809 005 (86.7)	1 141 389 (84.4)
<1	90 979 (9.8)	127 088 (9.4)
≥1	32 653 (3.5)	84 660 (6.3)
Age at menopause, mean (SD), y	NA	49.5 (4.3)
Age at menopause, y		
<40	NA	24 180 (1.8)
40-44	NA	79 572 (5.9)
45-49	NA	370 078 (27.3)
50-54	NA	738 106 (54.5)
≥55	NA	141 201 (10.4)
Total reproductive span, mean (SD), y	NA	32.9 (4.6)
Total reproductive span, y		
<30	NA	192 336 (14.2)
30 to <35	NA	567 528 (41.9)
35 to <40	NA	510 325 (37.7)
≥40	NA	82 948 (6.1)
HRT use, y	NA	
None	NA	1 140 253 (84.3)
<2	NA	124 150 (9.2)
2 to <5	NA	50 432 (3.7)
≥5	NA	38 302 (2.8)

Abbreviations: BMI, body mass index (calculated as weight in kilograms divided by height in meters squared); DBP, diastolic blood pressure; HRT, hormone replacement therapy; NA, not applicable; OC, oral contraceptive; SBP, systolic blood pressure.

SI conversion factors: To convert glucose to millimoles per liter, multiply by 0.0555; total cholesterol to millimoles per liter, multiply by 0.0259.

### Associations by Menopausal Status

**Figure 2.**
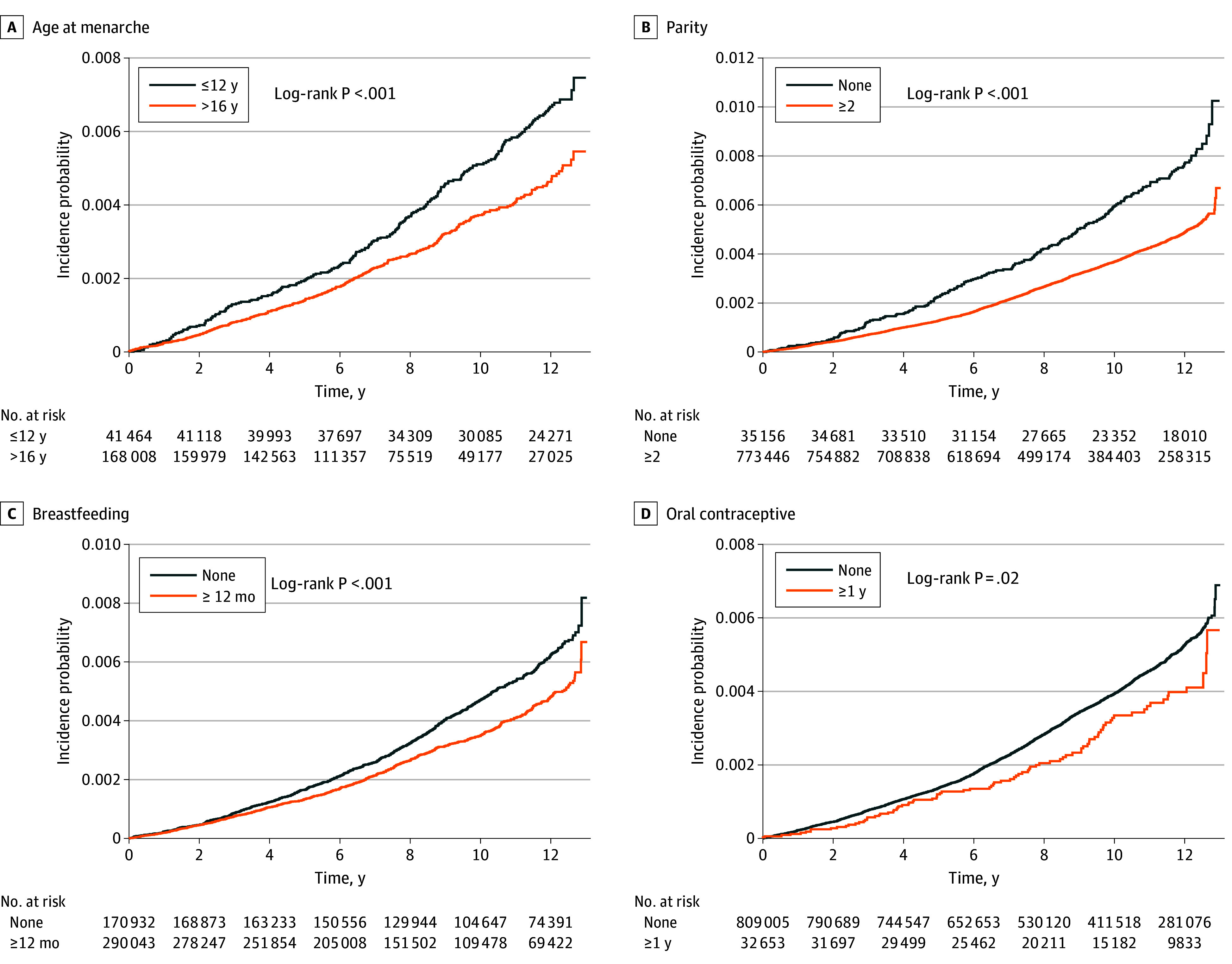
Survival Plot of the Incidence Probabilities of Ovarian Cancer in Premenopausal Women Kaplan-Meier curves show incidence by reproductive factors (age at menarche, parity, breastfeeding, oral contraceptive [OC] use) and were plotted after adjusting for age, income, smoking, drinking, exercise, comorbidities, and reproductive factors.

During a mean (SD) follow-up duration of 8.98 (3.33) years in premenopausal women and 11.87 (2.00) years in postmenopausal women, 3354 and 7375 incident ovarian cancer cases were observed, respectively. Cumulative incidence curves for premenopausal women are shown in Figure 2, and corresponding curves for postmenopausal women are presented in eFigure 1 in Supplement 1. In the fully adjusted model (model 4), earlier menarche (at age ≤12 years vs >16 years) was associated with higher ovarian cancer risk in both premenopausal women (HR, 1.37; 95% CI, 1.16-1.61) and postmenopausal women (HR, 1.24; 95% CI, 1.00-1.54). Parity of 2 or more live births was associated with lower risk in both premenopausal and postmenopausal groups (HR, 0.68 [95% CI, 0.58-0.79] and 0.71 [95% CI, 0.60-0.85]) (Table 2). Breastfeeding duration of 12 months or longer (HR, 0.86; 95% CI, 0.77-0.96) and OC use of 1 year or longer (HR, 0.75; 95% CI, 0.61-0.93) were associated with reduced risk only in premenopausal women, with no material association in postmenopausal women. Among postmenopausal women, later menopause (at age ≥55 years) and longer reproductive span (≥40 years) were associated with increased risk (HR, 1.36 [95% CI, 1.11-1.66] and 1.21 [95% CI, 1.09-1.34]). HRT use for 2 to 5 years was also associated with higher risk (HR, 1.20; 95% CI, 1.07-1.34).

**Table 2.** Multivariable-Adjusted Hazard Ratios of Ovarian Cancer by Reproductive Factors, Stratified by Menopausal Status

Reproductive factor	Premenopausal women			Postmenopausal women		
	Events, No.	HR (95% CI)^a^	*P* value^b^	Events, No.	HR (95% CI)^a^	*P* value^b^
Age at menarche, y						
≤12	233	1 [Reference]	.002	84	1 [Reference]	.01
13-14	1214	0.81 (0.70-0.93)		948	0.90 (0.72-1.12)	
15-16	1473	0.78 (0.68-0.89)		2860	0.84 (0.68-1.04)	
>16	434	0.73 (0.62-0.86)		3483	0.81 (0.65-1.00)	
Parity						
0	224	1 [Reference]	<.001	183	1 [Reference]	<.001
1	516	0.78 (0.66-0.93)		473	0.78 (0.65-0.94)	
≥2	2614	0.68 (0.58-0.79)		6719	0.71 (0.60-0.85)	
Breastfeeding duration, mo						
None	809	1 [Reference]	.05	556	1 [Reference]	.16
<6	911	0.92 (0.84-1.03)		482	1.00 (0.87-1.14)	
6 to <12	816	0.89 (0.80-0.99)		1195	0.91 (0.81-1.02)	
≥12	818	0.86 (0.77-0.96)		5142	0.92 (0.82-1.03)	
OC, y						
None	2928	1 [Reference]	.03	6218	1 [Reference]	.58
<1	339	1.01 (0.90-1.13)		705	1.01 (0.93-1.09)	
≥1	87	0.75 (0.61-0.93)		452	0.95 (0.86-1.05)	
Age at menopause, y						
<40	NA	NA	NA	109	1 [Reference]	<.001
40-44	NA	NA	NA	407	1.14 (0.92-1.41)	
45-49	NA	NA	NA	1865	1.12 (0.93-1.36)	
50-54	NA	NA	NA	4118	1.24 (1.03-1.51)	
≥55				876	1.36 (1.11-1.66)	
Total reproductive span, y						
<30	NA	NA	NA	986	1 [Reference]	<.001
30 to <35	NA	NA	NA	2882	1.00 (0.93-1.07)	
35 to <40	NA	NA	NA	2982	1.14 (1.06-1.23)	
≥40	NA	NA	NA	525	1.21 (1.09-1.34)	
HRT use, y						
None	NA	NA	NA	6185	1 [Reference]	.004
<2	NA	NA	NA	636	0.94 (0.86-1.02)	
2 to <5	NA	NA	NA	331	1.20 (1.07-1.34)	
≥5	NA	NA	NA	223	1.05 (0.92-1.20)	

Abbreviations: HR, hazard ratio; HRT, hormone replacement therapy; NA, not applicable; OC, oral contraceptive.

^a^ HR were adjusted for age, income level, smoking status, alcohol consumption, regular exercise, diabetes, hypertension, dyslipidemia, chronic kidney disease, and all reproductive factors (age at menarche, parity, breastfeeding duration, OC use, age at menopause, total reproductive span, and HRT use).

^b^ *P* values were calculated using Cox proportional hazards regression models.

### Variation by Birth Cohort

**Figure 3.**
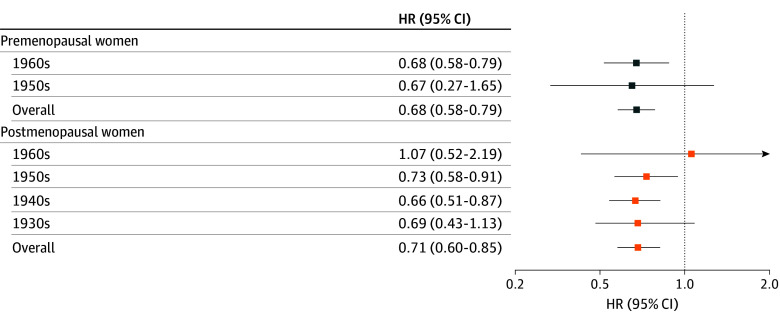
Forest Plot for Ovarian Cancer by Parity (≥2 vs None) Across Birth Cohorts, Stratified by Menopausal Status Points indicate hazard ratios (HRs), and error bars indicate 95% CIs.

Variation by birth cohort is summarized in eTables 1 and 2 in Supplement 1. Among premenopausal women, earlier menarche was associated with higher ovarian cancer risk in both the 1950s and 1960s cohorts, with a larger HR observed in those born in the 1950s than those born in the 1960s (HR, 3.45 [95% CI, 1.67-7.14] vs 1.32 [95% CI, 1.11-1.56]; *P* for interaction = .06). In the 1960s cohort, parity of 2 or more births (HR, 0.68; 95% CI, 0.58-0.79), breastfeeding duration of 12 months or longer (HR, 0.86; 95% CI, 0.77-0.96), and OC use for 1 year or longer (HR, 0.76; 95% CI, 0.61-0.94) were each associated with lower ovarian cancer risk (Figure 3; eTable 1 in Supplement 1). Corresponding estimates in the 1950s cohort were not statistically significant (parity: HR, 0.67 [95% CI, 0.27-1.65]; breastfeeding duration: HR, 0.88 [95% CI, 0.53-1.45]; OC use: HR, 0.68 [95% CI, 0.25-1.84]). Among postmenopausal women, earlier menarche showed higher HRs in the 1930s cohort (HR, 1.56; 95% CI, 0.78-3.13) and 1940s cohort (HR, 1.45; 95% CI, 0.97-2.17), with attenuated associations in later cohorts (*P* for interaction = .08). Parity of 2 or more births was associated with lower ovarian cancer risk in the 1930s (HR, 0.69; 95% CI, 0.43-1.13), 1940s (HR, 0.66; 95% CI, 0.51-0.87), and 1950s (HR, 0.73; 95% CI, 0.58-0.91) cohorts but not in the 1960s cohort (HR, 1.07; 95% CI, 0.52-2.19; *P* for interaction = .36) (Figure 3). Later menopause was associated with higher ovarian cancer risk in the 1940s cohort (HR, 1.65; 95% CI, 1.17-2.34) and 1950s cohort (HR, 1.33; 95% CI, 0.85-2.08) (eTable 2 in Supplement 1). Use of HRT for 2 to 5 years was associated with a higher ovarian cancer risk in the 1950s cohort (HR, 1.20; 95% CI, 1.02-1.40), with no significant associations in other cohorts (*P* for interaction = .69).

### Interactions Between Reproductive Factors

Exploratory analyses (eTables 3 and 4 in Supplement 1) showed no statistically significant interactions between reproductive factors in either premenopausal or postmenopausal women. The associations of parity, breastfeeding duration, and OC use were generally consistent across strata of age at menarche and other reproductive factors. No combination of factors materially altered the primary findings.

## Discussion

This nationwide cohort of 2 285 774 women in Korea—one of the largest single-country studies to date—provides robust evidence that reproductive factors historically associated with ovarian cancer risk show meaningful heterogeneity by menopausal status and birth cohort in the context of one of the world’s most rapid fertility transitions. Early menarche, later menopause, longer reproductive span, and HRT use were associated with higher risk, whereas higher parity, breastfeeding duration, and OC use were associated with lower risk. These findings align with established hormonal and ovulatory mechanisms^5,6,7,8,9^ and extend prior evidence by demonstrating that the magnitude and direction of these associations vary across birth cohorts.^1,2,3,4,10,11,24,25,26,27,28^

### Variation by Menopausal Status and Birth Cohort

Early menarche was associated with higher ovarian cancer risk in both premenopausal and postmenopausal women, consistent with longer lifetime hormonal exposure.^29,30,31,32,33^ Parity was associated with lower risk in both groups, in line with findings from large pooled and international cohorts.^10,11,24,25,26,34^ In contrast, breastfeeding duration of 12 months or longer and OC use of 1 year or longer were each associated with reduced risk only among premenopausal women; no significant associations were observed in postmenopausal women. This difference may reflect more recent exposure; shorter latency to diagnosis^15,16,35^; and, particularly for OC, generational differences in timing, formulation, cumulative duration, and lower lifetime exposure in older Asian populations.^10,11,12,13,26,36^ In postmenopausal women, later menopause, longer reproductive span, and HRT use (particularly 2-5 years) were associated with higher risk, consistent with prolonged ovulatory and estrogen exposure.^5,6,7,8,9,17,18^ Overall, these findings reinforce the role of lifetime ovulatory and hormonal exposures in ovarian cancer etiological factors, with risk patterns differing by menopausal status and timing of exposure.^37^

The risk-lowering association for parity was greater in magnitude in earlier postmenopausal cohorts (1930s to 1950s), whereas no meaningful association was observed in the 1960s cohort. This pattern aligns with Korea’s rapid fertility decline and similar rates in other low-fertility countries.^12,13,14,19,20,21,38,39,40^ In high-fertility generations, parity of 2 or more often meant 4 or more births, creating a wide exposure contrast with nulliparous women. In more recent low-fertility cohorts, the same parity category mostly reflected 2 to 3 births, narrowing the exposure gradient and reducing the magnitude of the association despite higher lifetime ovulatory cycles in these generations. Age, period, and cohort analyses and global burden analyses have reported comparable generational shifts in East Asia and Europe.^12,13,14,26,38,39,40^ The present study adds large-scale individual-level evidence from a population undergoing one of the world’s most rapid fertility transitions,^19,21,41^ complementing prior consortia that were predominantly based on Western cohorts.^10,11,25,27^

### Implications of Fertility Decline

eFigure 2 in Supplement 1 shows an ecological pattern in Korea: as total fertility rates have sharply declined over the past decades, ovarian cancer incidence has increased with an approximate 30- to 40-year lag—a pattern consistent with previous studies in Japan, Southern Europe, and Nordic countries.^12,13,14,20,21,22,38,39,40^ In the present study, higher parity was associated with lower HRs mainly in earlier, high-fertility cohorts, whereas these associations were attenuated or absent in recent, low-fertility cohorts. As national fertility continues to decline, future generations may lack the parity-related reduction in risk observed in older cohorts. Although ecological patterns do not establish individual-level causal association, they suggest that demographic shifts could alter population-level ovarian cancer risk distributions over time. Continued monitoring of reproductive patterns and ovarian cancer incidence is warranted in aging, low-fertility societies. In the absence of effective population-based screening for ovarian cancer,^42^ continued refinement of risk assessment and improved understanding of reproductive and hormonal patterns^25,43^ and implementation of risk-reducing strategies for high-risk women^44^ remain critical public health priorities.^1,39^

### Strengths and Limitations

This study has several strengths. Its large population and linkage to the national cancer registry and rare disease programs allowed for near-complete case ascertainment.^4^ The availability of standardized, populationwide reproductive and health examination data from the NHIS enabled the detailed assessment of life-course reproductive factors.^45,46^ In addition, generational stratification and interaction testing provided insights into temporal shifts that would not be detectable in smaller cohorts. Use of validated administrative and screening data, along with established analytic methods, including Cox proportional hazards regression models and Schoenfeld residual diagnostics, supported internal validity. The NHIS infrastructure, which has enabled numerous large-scale epidemiologic investigations, also enhances generalizability to other settings with universal health coverage.^45,46^

Study limitations include self-reported reproductive variables that were prone to recall bias (especially in older women), lack of histologic subtype data,^25^ absence of key confounders (tubal ligation, family history, and *BRCA1/2* status),^47,48^ lower OC exposure and fewer events in older cohorts,^10,11,15,36^ optional questionnaire items leading to missing data with potential selection bias, and the inherent inability of observational studies to establish causal association or fully exclude residual confounding. Despite these limitations, the overall patterns observed in this cohort are directionally consistent with findings from prior biologic,^5,6,7,8,9^ epidemiologic, and pooled cohort studies across diverse populations.^12,13,14,24,38,39^

## Conclusions

In this cohort study, early menarche, later menopause, and longer reproductive span were associated with higher ovarian cancer risk, whereas parity was associated with lower risk across the premenopausal and postmenopausal groups. Breastfeeding duration and OC use were associated with lower risk only in premenopausal women. The risk-lowering association for parity was attenuated in the 1960s birth cohort in the context of Korea’s rapid fertility decline. These findings highlight meaningful differences in reproductive risk patterns across menopausal status and birth cohorts, with implications for risk stratification in low-fertility, aging populations. Further research that incorporates histologic subtypes, genetic factors, and detailed exposure histories is needed.

## Notes

Supplement 1. **eTable 1.** Multivariable-Adjusted Hazard Ratios of Ovarian Cancer by Reproductive Factors and Birth Cohort in Premenopausal Women **eTable 2.** Multivariable-Adjusted Hazard Ratios of Ovarian Cancer by Reproductive Factors and Birth Cohort in Postmenopausal Women **eTable 3.** Multivariable-Adjusted Hazard Ratios for Interactions Between Reproductive Factors in Premenopausal Women **eTable 4.** Multivariable-Adjusted Hazard Ratios for Interactions Between Reproductive Factors in Postmenopausal Women **eFigure 1.** Kaplan-Meier Curves for Incidence Probabilities of Ovarian Cancer in Postmenopausal Women **eFigure 2.** Trends in Total Fertility Rate (1972–1981) and Ovarian Cancer Incidence (2012–2021) in Korea

Supplement 2.
Data Sharing Statement
