# Supplementary material for: Reproductive Shifts and Ovarian Cancer Risk in Women Aged 40 Years or Older
Source: JAMA Netw Open. 2026 Feb 3;9(2):e2556840. doi: 10.1001/jamanetworkopen.2025.56840 (PMC12869340; doi:10.1001/jamanetworkopen.2025.56840)
Supplement: Supplement 1. — eTable 1. Multivariable-Adjusted Hazard Ratios of Ovarian Cancer by Reproductive Factors and Birth Cohort in Premenopausal Women eTable 2. Multivariable-Adjusted Hazard Ratios of Ovarian Cancer by Reproductive Factors and Birth Cohort in Postmenopausal Women eTable 3. Multivariable-Adjusted Hazard Ratios for Interactions Between Reproductive Factors in Premenopausal Women eTable 4. Multivariable-Adjusted Hazard Ratios for Interactions Between Reproductive Factors in Postmenopausal Women eFigure 1. Kaplan-Meier Curves for Incidence Probabilities of Ovarian Cancer in Postmenopausal Women eFigure 2. Trends in Total Fertility Rate (1972–1981) and Ovarian Cancer Incidence (2012–2021) in Korea [file jamanetwopen-e2556840-s001.pdf]

Supplemental Online Content

Kim JH, Hwang IS, Lee SJ, Kim CJ, Lee SJ, Han K. Reproductive shifts and ovarian cancer risk in women. *JAMA Netw Open*. 2026;9(2):e2556840. doi:10.1001/jamanetworkopen.2025.56840

eTable 1. Multivariable-Adjusted Hazard Ratios of Ovarian Cancer by Reproductive Factors and Birth Cohort in Premenopausal Women

eTable 2. Multivariable-Adjusted Hazard Ratios of Ovarian Cancer by Reproductive Factors and Birth Cohort in Postmenopausal Women

eTable 3. Multivariable-Adjusted Hazard Ratios for Interactions Between Reproductive Factors in Premenopausal Women

eTable 4. Multivariable-Adjusted Hazard Ratios for Interactions Between Reproductive Factors in Postmenopausal Women

eFigure 1. Kaplan-Meier Curves for Incidence Probabilities of Ovarian Cancer in Postmenopausal Women

eFigure 2. Trends in Total Fertility Rate (1972–1981) and Ovarian Cancer Incidence (2012–2021) in Korea

This supplemental material has been provided by the authors to give readers additional information about their work.

© 2026 Kim JH et al. *JAMA Network Open*

eTable 1: Multivariable-Adjusted Hazard Ratios of Ovarian Cancer by Reproductive Factors and Birth Cohort in Premenopausal Women

	1950s		1960s		
Reproductive Factor	Events, No.	HR[a] (95%CI)	Events, No.	HR[a] (95%CI)	P for interaction
Age at menarche					
≤12 years	9	1 [Reference]	224	1 [Reference]	0.06
13-14 years	36	0.42 (0.20-0.88)	1178	0.82 (0.71-0.95)	
15-16 years	65	0.34 (0.17-0.69)	1408	0.80 (0.69-0.92)	
>16 years	37	0.29 (0.14-0.60)	397	0.76 (0.64-0.90)	
Parity					
0	5	1 [Reference]	219	1 [Reference]	0.86
1	23	0.88(0.33-2.32)	493	0.78 (0.66-0.93)	
≥2	119	0.67(0.27-1.65)	2495	0.68 (0.58-0.79)	
Breast Feeding					
None	20	1 [Reference]	789	1 [Reference]	0.97
<6 months	20	0.84 (0.45-1.57)	891	0.93 (0.84-1.03)	
6–12 months	40	0.90 (0.52-1.54)	776	0.89 (0.80-1.00)	
≥12 months	67	0.88 (0.53-1.45)	751	0.86 (0.77-0.96)	
Oral Contraceptive Use					
None	126	1 [Reference]	2802	1 [Reference]	0.78
<1 year	17	1.20 (0.72-1.98)	322	1.00 (0.89-1.13)	
≥1 year	4	0.68 (0.25-1.84)	83	0.76 (0.61-0.94)	

Abbreviation: IR, Incidence rate per 1,000 person-years; HR, Hazard ratio; CI, Confidence interval; NA, Not applicable. aHR were adjusted for age, income, smoking, drinking, regular exercise, diabetes mellitus, hypertension, dyslipidemia, chronic kidney disease, and all reproductive factors (age at menarche, parity, breastfeeding, OC use, age at menopause, total reproductive span, hormone replacement therapy). P-values were calculated using Cox proportional hazards models.

© 2026 Kim JH et al. *JAMA Network Open*

eTable 2: Multivariable-Adjusted Hazard Ratios of Ovarian Cancer by Reproductive Factors and Birth Cohort in Postmenopausal Women

	~1930s		1940s		1950s		1960s		
	Events,		Events,		Events,		Events,		*P* for
Reproductive Factor	No.	HR (95%CI)	No.	HR (95%CI)	No.	HR (95%CI)	No.	HR (95%CI)	interaction
Age at menarche									
≤12 years	8	1 [Reference]	24	1 [Reference]	44	1 [Reference]	8	1 [Reference]	0.08
13-14 years	126	0.91 (0.45-1.86)	278	0.74 (0.49-1.12)	496	0.97 (0.72-1.32)	48	0.78 (0.37-1.65)	
15-16 years	485	0.66 (0.33-1.33)	1019	0.71 (0.47-1.06)	1271	0.94 (0.69-1.27)	85	0.84 (0.41-1.74)	
>16 years	866	0.64 (0.32-1.28)	1475	0.69 (0.46-1.03)	1083	0.87 (0.65-1.18)	59	1.10 (0.53-2.32)	
Parity									
0	17	1 [Reference]	62	1 [Reference]	96	1 [Reference]	8	1 [Reference]	0.36
1	42	0.87 (0.49-1.54)	141	0.83 (0.61-1.14)	260	0.74 (0.56-0.94)	30	1.01 (0.46-2.21)	
≥2	1426	0.69 (0.43-1.13)	2593	0.66 (0.51-0.87)	2538	0.73 (0.58-0.91)	162	1.07 (0.52-2.19)	
Breast Feeding									
None	41	1 [Reference]	171	1 [Reference]	305	1 [Reference]	39	1 [Reference]	0.78
<6 months	28	0.84 (0.52-1.36)	119	0.88 (0.69-1.13)	294	1.06 (0.90-1.26)	41	1.12 (0.72-1.73)	
6–12 months	147	0.94 (0.66-1.34)	396	0.88 (0.73-1.07)	600	0.90 (0.78-1.05)	52	1.02 (0.67-1.55)	
≥12 months	1269	0.98 (0.71-1.34)	2110	0.84 (0.71-1.00)	1695	0.94 (0.82-1.08)	68	0.92 (0.62-1.37)	
Oral Contraceptive Use									
None	1325	1 [Reference]	2290	1 [Reference]	2436	1 [Reference]	167	1 [Reference]	0.28
<1 year	77	0.79 (0.63-0.99)	296	1.00 (0.88-1.13)	308	1.08 (0.96-1.21)	24	1.23 (0.80-1.89)	
≥1 year	83	1.04 (0.83-1.30)	210	0.93 (0.81-1.07)	150	0.93 (0.79-1.10)	9	1.01 (0.52-1.99)	
Age at Menopause									
<40	44	1 [Reference]	34	1 [Reference]	21	1 [Reference]	10	1 [Reference]	0.36
40-44	141	1.03 (0.74-1.45)	147	1.46 (1.01-2.12)	85	1.04 (0.65-1.68)	34	0.88 (0.44-1.78)	
45-49	417	1.00 (0.73-1.36)	627	1.37 (0.97-1.93)	665	1.04 (0.68-1.61)	156	1.16 (0.61-2.20)	
50-54	735	1.00 (0.74-1.35)	1482	1.51 (1.07-2.12)	1901	1.21 (0.78-1.86)	NA	NA	
≥55	148	1.05 (0.75-1.46)	506	1.65 (1.17-2.34)	222	1.33 (0.85-2.08)	NA	NA	
Total Reproductive Span									
<30 years	356	1 [Reference]	348	1 [Reference]	218	1 [Reference]	64	1 [Reference]	0.25

© 2026 Kim JH et al. *JAMA Network Open*

30-35 years	633	0.85 (0.75-0.97)	1052	1.07 (0.95-1.21)	1071	1.03 (0.89-1.19)	126	1.15 (0.85-1.55)	
35-40 years	424	1.03 (0.89-1.18)	1101	1.19 (1.05-1.34)	1447	1.17 (1.01-1.35)	10	0.97 (0.50-1.90)	
≥40 years	72	0.91 (0.705,1.17)	295	1.32 (1.13-1.54)	158	1.24 (1.01-1.53)	0	NA	
Hormone Replacement Therapy									
None	1401	1 [Reference]	2282	1 [Reference]	2343	1 [Reference]	159	1 [Reference]	0.69
<2 years	49	1.07 (0.81-1.43)	251	1.01 (0.88-1.15)	308	0.88 (0.78-0.99)	28	0.81 (0.54-1.21)	
2–5 years	21	1.22 (0.79-1.87)	133	1.18 (0.99-1.41)	166	1.20 (1.02-1.40)	11	1.10 (0.60-2.03)	
≥5 years	14	0.82 (0.49-1.39)	130	1.12 (0.94-1.34)	77	1.00 (0.79-1.25)	2	0.50 (0.12-2.00)	

Abbreviation: IR, Incidence rate per 1,000 person-years; HR, Hazard ratio; CI, Confidence interval; NA, Not applicable. aHR were adjusted for age, income, smoking, drinking, regular exercise, diabetes mellitus, hypertension, dyslipidemia, chronic kidney disease, and all reproductive factors (age at menarche, parity, breastfeeding, OC use, age at menopause, total reproductive span, hormone replacement therapy). P-values were calculated using Cox proportional hazards models.

© 2026 Kim JH et al. *JAMA Network Open*

eTable 3: Multivariable-Adjusted Hazard Ratios for Interactions Between Reproductive Factors in Premenopausal Women

Reproductive Factor 1		Reproductive Factor 2		Events, No.	aHR (95%CI)	*P* value	bHR (95%CI)	*P* for interaction
Age at menarche ≥15	No	Parity	No	125	1 [Reference]	<.001	1 [Reference]	0.22
			Yes	1322	0.65 (0.53-0.79)		0.65 (0.53-0.79)	
	Yes		No	99	0.78 (0.60-1.02)		1 [Reference]	
			Yes	1808	0.60 (0.50-0.73)		0.77 (0.62-0.96)	
Age at menarche ≥15	No	Breast Feeding	No	401	1 [Reference]	0.003	1 [Reference]	0.93
			Yes	1046	0.88 (0.78-1.00)		0.88 (0.78-1.00)	
	Yes		No	408	0.92 (0.80-1.06)		1 [Reference]	
			Yes	1499	0.81 (0.72-0.91)		0.88 (0.78-0.99)	
Age at menarche ≥15	No	OC	No	1246	1 [Reference]	0.028	1 [Reference]	0.09
			Yes	201	1.05 (0.90-1.22)		1.05 (0.90-1.22)	
	Yes		No	1682	0.94 (0.87-1.01)		1 [Reference]	
			Yes	225	0.83 (0.72-0.96)		0.88 1(0.77-1.01)	
Parity	No	Breast Feeding	No	224	1 [Reference]	<.001	NA	<.001
	Yes		No	585	0.70 (0.60-0.82)		1 [Reference]	
			Yes	2545	0.62 (0.54-0.71)		0.88 (0.80-0.96)	
Parity	No	OC	No	197	1 [Reference]	<.001	1 [Reference]	0.69
			Yes	27	0.88 (0.59-1.32)		0.88 (0.59-1.32)	
	Yes		No	2731	0.70 (0.59-0.82)		1 [Reference]	
			Yes	399	0.67 (0.55-0.80)		0.96 (0.86-1.07)	
Breast Feeding	No	OC	No	707	1 [Reference]	0.03	1 [Reference]	0.64
			Yes	102	0.91 (0.74-1.13)		0.91 (0.74-1.13)	
	Yes		No	2221	0.87 (0.79-0.96)		1 [Reference]	
			Yes	324	0.84 (0.73-0.97)		0.97 (0.86-1.09)	

Abbreviation: HR = hazard ratio; CI = confidence interval; OC = oral contraceptive. Model 4 adjusted for age, income, smoking, alcohol consumption, physical activity, diabetes, hypertension, dyslipidemia, chronic kidney disease, and all reproductive factors. aHR (95% CI): Stratum-specific association with Factor 1 (reference: both factors absent). bHR (95% CI): Association from model with Factor 1 × Factor 2 interaction term (reference: Factor 2 absent when Factor 1 present; Factor 1 absent when Factor 2 present). Events = incident ovarian cancer cases in premenopausal women.

© 2026 Kim JH et al. *JAMA Network Open*

eTable 4: Multivariable-Adjusted Hazard Ratios for Interactions Between Reproductive Factors in Postmenopausal Women

Reproductive Factor 1		Reproductive Factor 2		Events, No.	[a]HR (95%CI)	*P* value	[b]HR (95%CI)	*P* for interaction
Age at menarche ≥15	No	Parity	No	42	1 [Reference]	<.001	1 [Reference]	0.76
			Yes	990	0.77 (0.55–1.06)		0.77 (0.55–1.06)	
	Yes	Parity	No	141	0.96 (0.68–1.35)		1 [Reference]	
			Yes	6202	0.69 (0.50–0.95)		0.72 (0.60–0.88)	
Age at menarche ≥15	No	Breast Feeding	No	139	1 [Reference]	0.007	1 [Reference]	0.77
			Yes	893	0.88 (0.73–1.07)		0.88 (0.73–1.07)	
	Yes	Breast Feeding	No	417	0.88 (0.73–1.07)		1 [Reference]	
			Yes	5926	0.81 (0.67–0.96)		0.91 (0.81–1.02)	
Age at menarche ≥15	No	OC	No	845	1 [Reference]	0.03	1 [Reference]	0.30
			Yes	187	1.07 (0.91–1.25)		1.07 (0.91–1.25)	
	Yes	OC	No	5373	0.92 (0.86–0.99)		1 [Reference]	
			Yes	970	0.90 (0.82–0.98)		0.97 (0.91–1.04)	
Age at menarche ≥15	No	Age at menopause	<40	14	1 [Reference]	0.007	1 [Reference]	0.88
			≥40	1018	1.26 (0.74–2.14)		1.26 (0.74–2.14)	
	Yes	Age at menopause	<40	95	0.95 (0.54–1.66)		1 [Reference]	
			≥40	6248	1.15 (0.68–1.94)		1.21 (0.99–1.48)	
Age at menarche ≥15	No	HRT	No	811	1 [Reference]	0.04	1 [Reference]	0.83
			Yes	221	1.03 (0.89–1.20)		1.03 (0.89–1.20)	
	Yes	HRT	No	5374	0.91 (0.84–0.98)		1 [Reference]	
			Yes	969	0.92 (0.84–1.01)		1.01 (0.95–1.09)	
Parity	No	Breast Feeding	No	183	1 [Reference]	<.001	NA	<.001
	Yes	Breast Feeding	No	373	0.73 (0.61–0.88)		1 [Reference]	
			Yes	6819	0.66 (0.57–0.77)		0.91 (0.81–1.00)	
Parity	No	OC	No	169	1 [Reference]	0.006	1 [Reference]	0.55
			Yes	14	0.83 (0.48–1.44)		0.83 (0.48–1.44)	
	Yes	OC	No	6049	0.72 (0.60–0.87)		1 [Reference]	
			Yes	1143	0.71 (0.59–0.86)		0.99 (0.93–1.05)	
Parity	No	Age at menopause	<40	0	1 [Reference]	0.001	NA	0.85
			≥40	183	NA		NA	

© 2026 Kim JH et al. *JAMA Network Open*

	Yes	Age at menopause	<40	109	NA		1 [Reference]	
			≥40	7083	NA		1.17 (0.97–1.42)	
Parity	No	HRT	No	139	1 [Reference]	0.007	1 [Reference]	0.97
			Yes	44	1.01 (0.72–1.42)		1.01 (0.72–1.42)	
	Yes	HRT	No	6046	0.73 (0.60–0.89)		1 [Reference]	
			Yes	1146	0.74 (0.61–0.91)		1.02 (0.95–1.09)	
Breast Feeding	No	OC	No	481	1 [Reference]	0.29	1 [Reference]	0.80
			Yes	75	1.02 (0.80–1.30)		1.02 (0.80–1.30)	
	Yes	OC	No	5737	0.91 (0.81–1.02)		1 [Reference]	
			Yes	1082	0.90 (0.79–1.01)		0.98 (0.92–1.05)	
Breast Feeding	No	Age at menopause	<40	9	1 [Reference]	0.05	1 [Reference]	0.80
			≥40	547	1.32 (0.68–2.55)		1.32 (0.68–2.55)	
	Yes	Age at menopause	<40	100	0.99 (0.50–1.96)		1 [Reference]	
			≥40	6719	1.19 (0.62–2.30)		1.21 (0.99–1.47)	
Breast Feeding	No	HRT	No	426	1 [Reference]	0.28	1 [Reference]	0.82
			Yes	130	1.04 (0.85–1.26)		1.04 (0.85–1.26)	
	Yes	HRT	No	5759	0.91 (0.81–1.02)		1 [Reference]	
			Yes	1060	0.92 (0.81–1.05)		1.01 (0.95–1.08)	
OC	No	Age at menopause	<40	92	1 [Reference]	0.21	1 [Reference]	0.61
			≥40	6126	1.24 (1.01–1.52)		1.24 (1.01–1.52)	1 [Reference]
	Yes	Age at menopause	<40	17	1.13 (0.67–1.89)		1 [Reference]	
			≥40	1140	1.22 (0.99–1.51)		1.08 (0.67–1.75)	
OC	No	HRT	No	5329	1 [Reference]	0.93	1 [Reference]	0.90
			Yes	889	1.02 (0.95–1.10)		1.02 (0.95–1.10)	
	Yes	HRT	No	856	0.99 (0.92–1.06)		1 [Reference]	
			Yes	301	1.00 (0.89–1.12)		1.01 (0.89–1.15)	
Age at menopause	<40	HRT	No	96	1 [Reference]	0.10	1 [Reference]	0.13
			Yes	13	0.65 (0.36–1.16)		0.65 (0.36–1.16)	
	≥40	HRT	No	6089	1.14 (0.93–1.39)		1 [Reference]	
			Yes	1177	1.17 (0.95–1.44)		1.02 (0.96–1.09)	
Total reproductive span	<30	Age at menopause	<40	55	1 [Reference]	0.009	1 [Reference]	0.65
	≥30		≥40	931	0.97 (0.74–1.27)		0.97 (0.74–1.27)	

© 2026 Kim JH et al. *JAMA Network Open*

	<30	Age at menopause	<40	977	1.13 (0.86–1.49)		1 [Reference]	
	≥30		≥40	5412	1.03 (0.79–1.34)		0.91 (0.85–0.97)	
Total reproductive span	<30	Parity	No	26	1 [Reference]	0.001	1 [Reference]	0.99
	≥30			960	0.73 (0.49–1.10)		0.73 (0.49–1.10)	
	<30		Yes	157	1.07 (0.71–1.63)		1 [Reference]	
	≥30			6232	0.79 (0.53–1.17)		0.73 (0.61–0.88)	
Total reproductive span	<30	Breast Feeding	No	66	1 [Reference]	0.03	1 [Reference]	0.38
	≥30			920	1.00 (0.77–1.29)		1.00 (0.77–1.29)	
	<30		Yes	490	1.20 (0.93–1.55)		1 [Reference]	
	≥30			5899	1.06 (0.82–1.36)		0.89 (0.79–0.99)	
Total reproductive span	<30	OC	No	834	1 [Reference]	0.20	1 [Reference]	0.56
	≥30			152	1.04 (0.87–1.23)		1.04 (0.87–1.23)	
	<30		Yes	5384	1.08 (1.00–1.16)		1 [Reference]	
	≥30			1005	1.06 (0.97–1.16)		0.98 (0.92–1.05)	
Total reproductive span	<30	Age at menopause	<40	109	1 [Reference]	0.10	1 [Reference]	0.91
	≥30			877	1.16 (0.95–1.42)		1.16 (0.95–1.42)	
	<30		≥40	0	NA		NA	
	≥30			6389	1.22 (1.01–1.48)		NA	
Total reproductive span	<30	HRT	No	843	1 [Reference]	0.12	1 [Reference]	0.24
	≥30		Yes	143	0.92 (0.77–1.10)		0.92 (0.77–1.10)	
	<30	HRT	No	5342	1.05 (0.98–1.13)		1 [Reference]	
	≥30		Yes	1047	1.09 (0.99–1.20)		1.03 (0.97–1.11)	

Abbreviation: HR = hazard ratio; CI = confidence interval; OC = oral contraceptive. Model 4 adjusted for age, income, smoking, alcohol consumption, physical activity, diabetes, hypertension, dyslipidemia, chronic kidney disease, and all reproductive factors. aHR (95% CI): Stratum-specific association with Factor 1 (reference: both factors absent). bHR (95% CI): Association from model with Factor 1 × Factor 2 interaction term (reference: Factor 2 absent when Factor 1 present; Factor 1 absent when Factor 2 present). Events = incident ovarian cancer cases in premenopausal women.

© 2026 Kim JH et al. *JAMA Network Open*

eFigure 1. Kaplan-Meier Curves for Incidence Probabilities of Ovarian Cancer in Postmenopausal Women

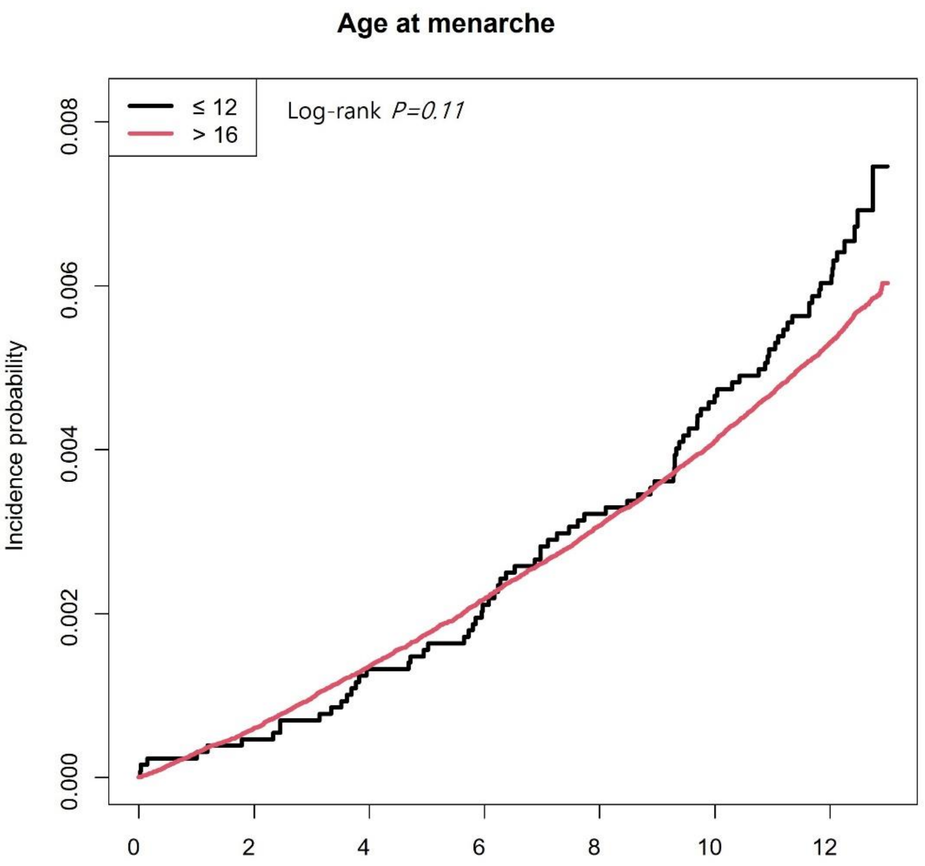

**No. at risk**							
Age at menarche							
≤12	12,926	12,871	12,791	12,662	12,537	12,365	12,173
>16	657,103	649,208	638,929	626,184	610,469	592,296	569,667

© 2026 Kim JH et al. *JAMA Network Open*

Parity

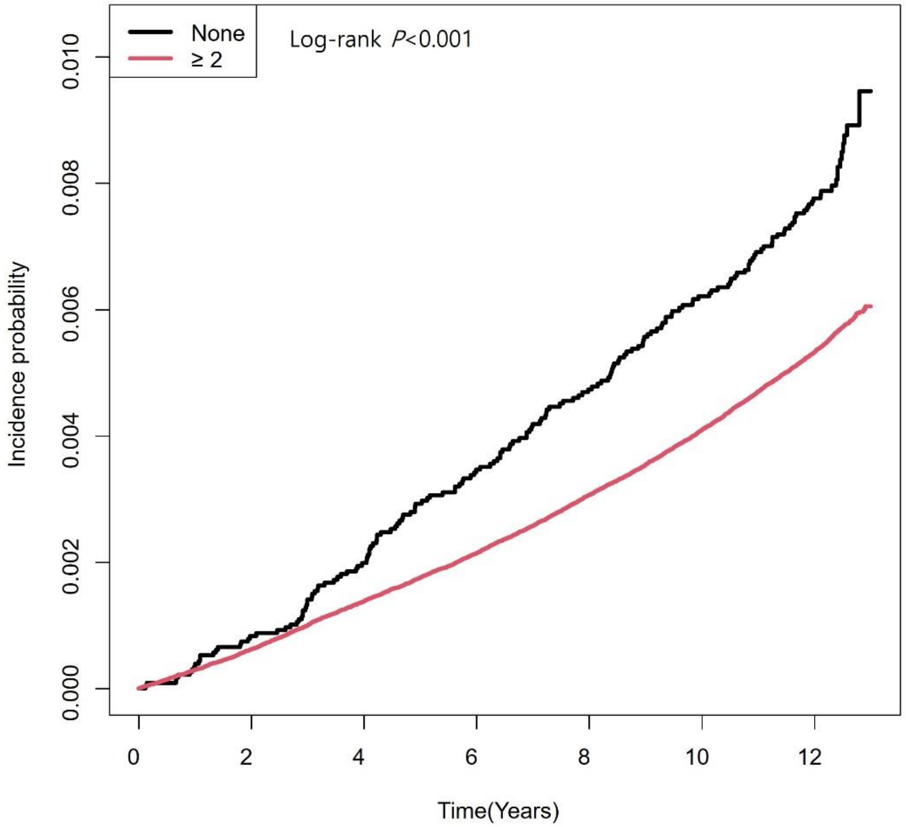

No. at risk

Parity							
None	22,800	22,620	22,374	22,117	21,780	21,409	20,945
≥2	1,250,351	1,237,520	1,220,775	1,199,673	1,173,676	1,143,697	1,106,235

© 2026 Kim JH et al. *JAMA Network Open*

Breast Feeding

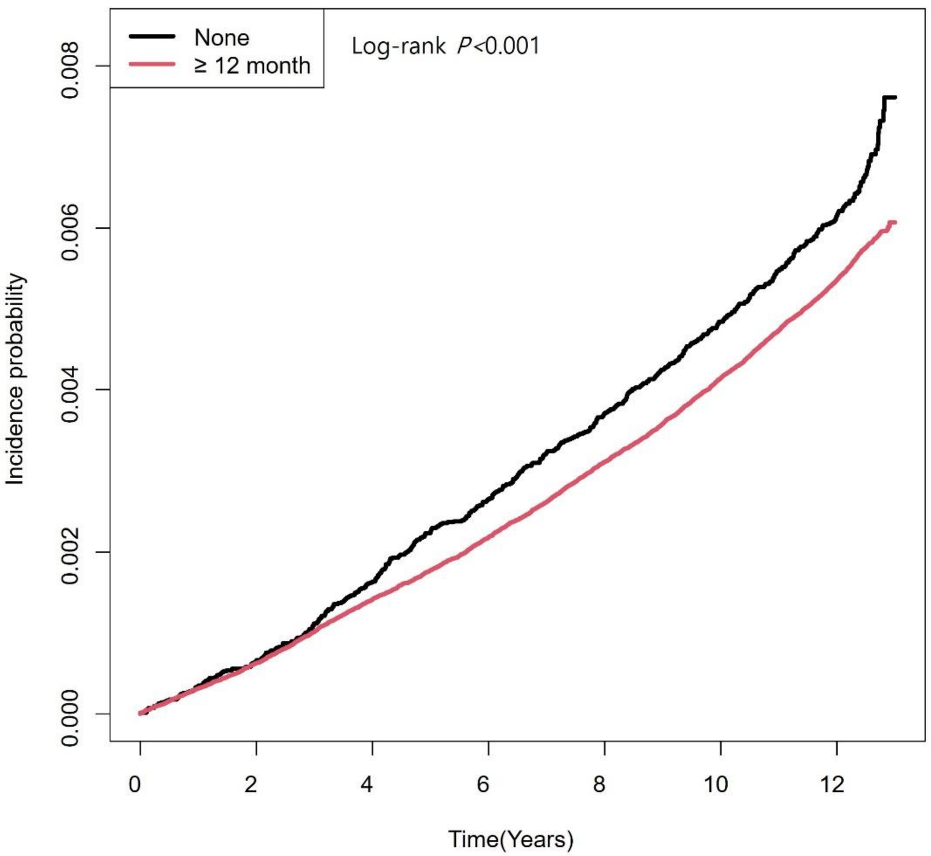

No. at risk

Breast Feeding							
None	86,200	85,688	84,980	84,192	83,168	82,024	80,550
≥12 months	957,569	946,230	931,934	913,774	891,378	865,595	833,443

© 2026 Kim JH et al. *JAMA Network Open*

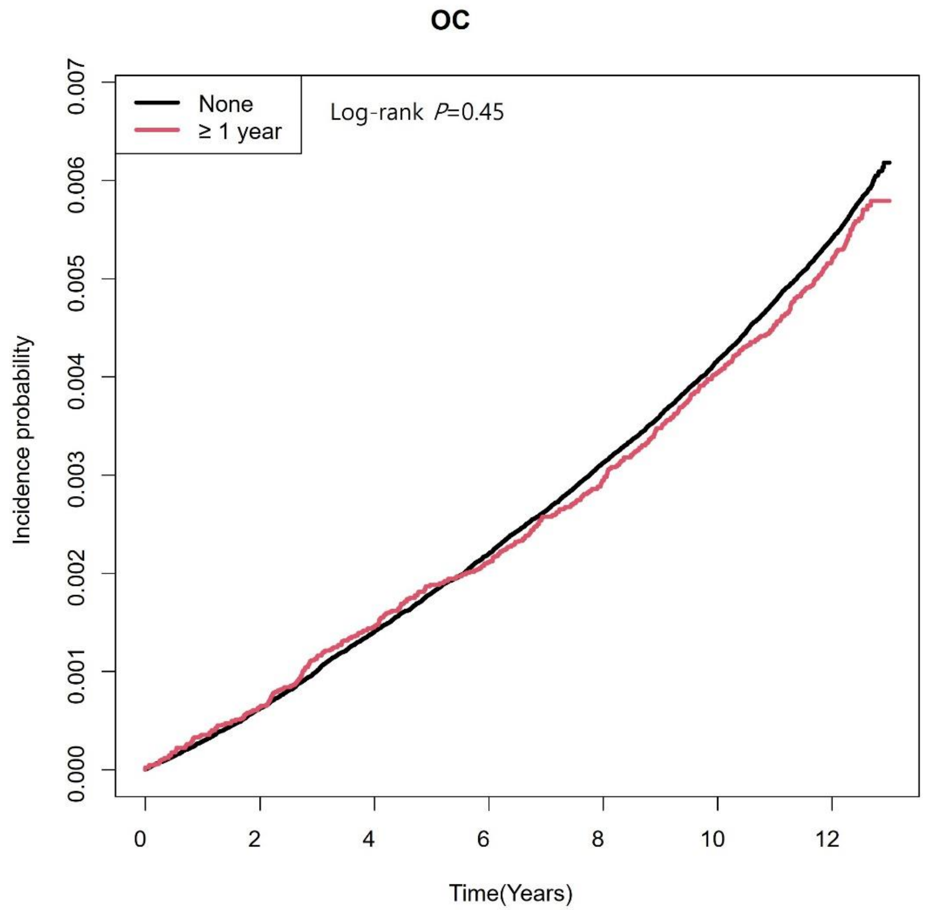

No. at risk							
OC							
Non	1,141,389	1,129,266	1,113,448	1,093,620	1,069,335	1,041,464	1,006,912
≥1 year	84,660	84,064	83,272	82,228	80,978	79,392	77,397

© 2026 Kim JH et al. *JAMA Network Open*

Age at menopause

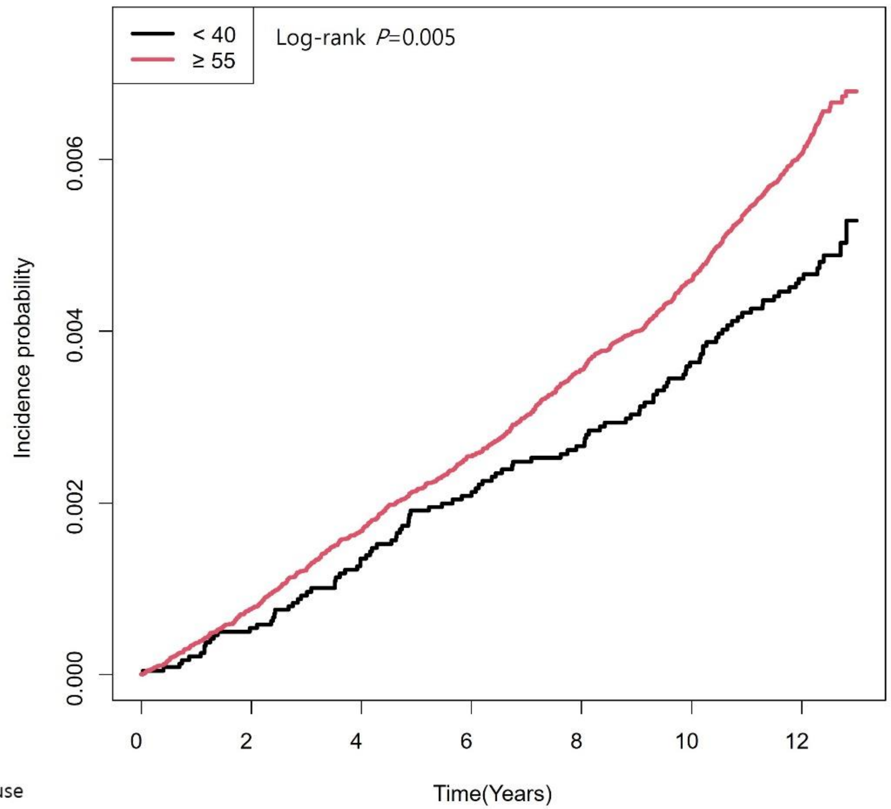

No. at risk

Age at menopause							
<40	24,180	23,798	23,327	22,700	21,908	20,963	19,833
≥55	141,201	140,001	138,414	136,375	133,894	130,756	126,836

© 2026 Kim JH et al. *JAMA Network Open*

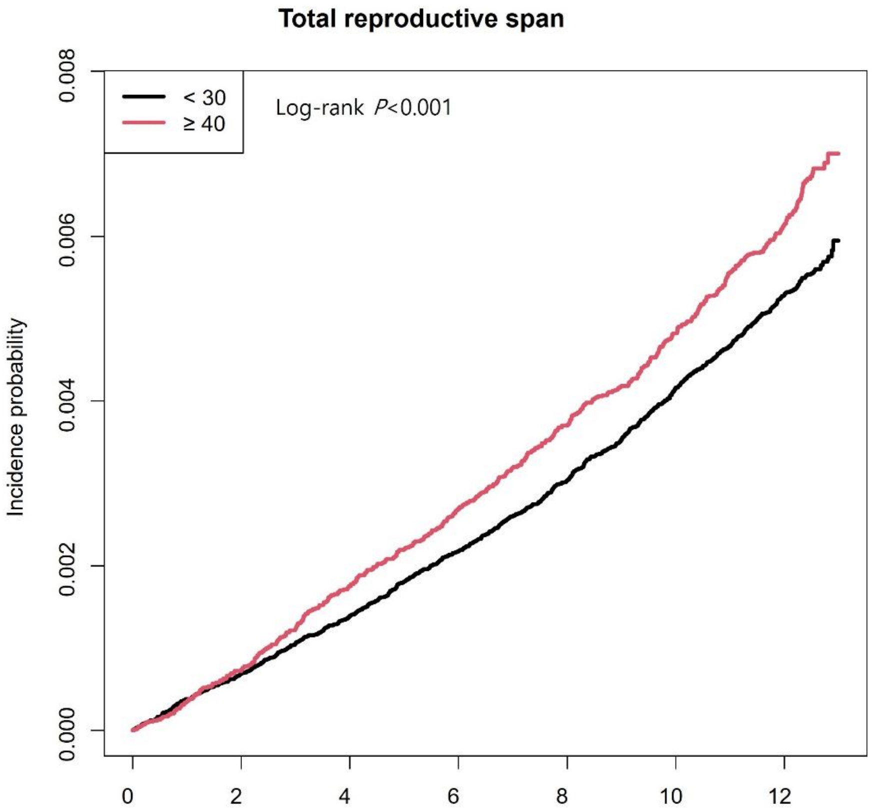

No. at risk

Total reproductive span							
<30	192,336	189,458	185,756	181,084	175,389	168,657	160,494
≥40	82,948	82,354	81,493	80,385	79,046	77,336	75,243

© 2026 Kim JH et al. *JAMA Network Open*

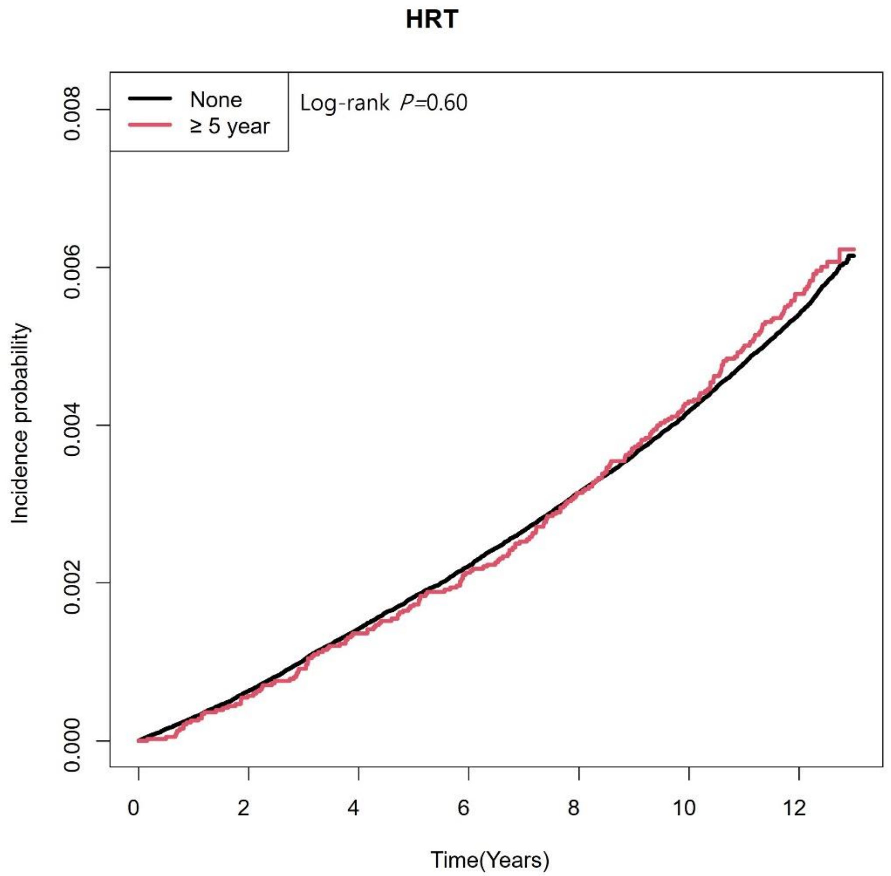

No. at risk

HRT							
None	1,140,253	1,127,597	1,111,099	1,090,422	1,064,922	1,035,695	999,295
≥5 years	38,302	38,143	37,934	37,624	37,265	36,811	36,206

© 2026 Kim JH et al. *JAMA Network Open*

eFigure 2. Trends in Total Fertility Rate (1972–1981) and Ovarian Cancer Incidence (2012–2021) in Korea

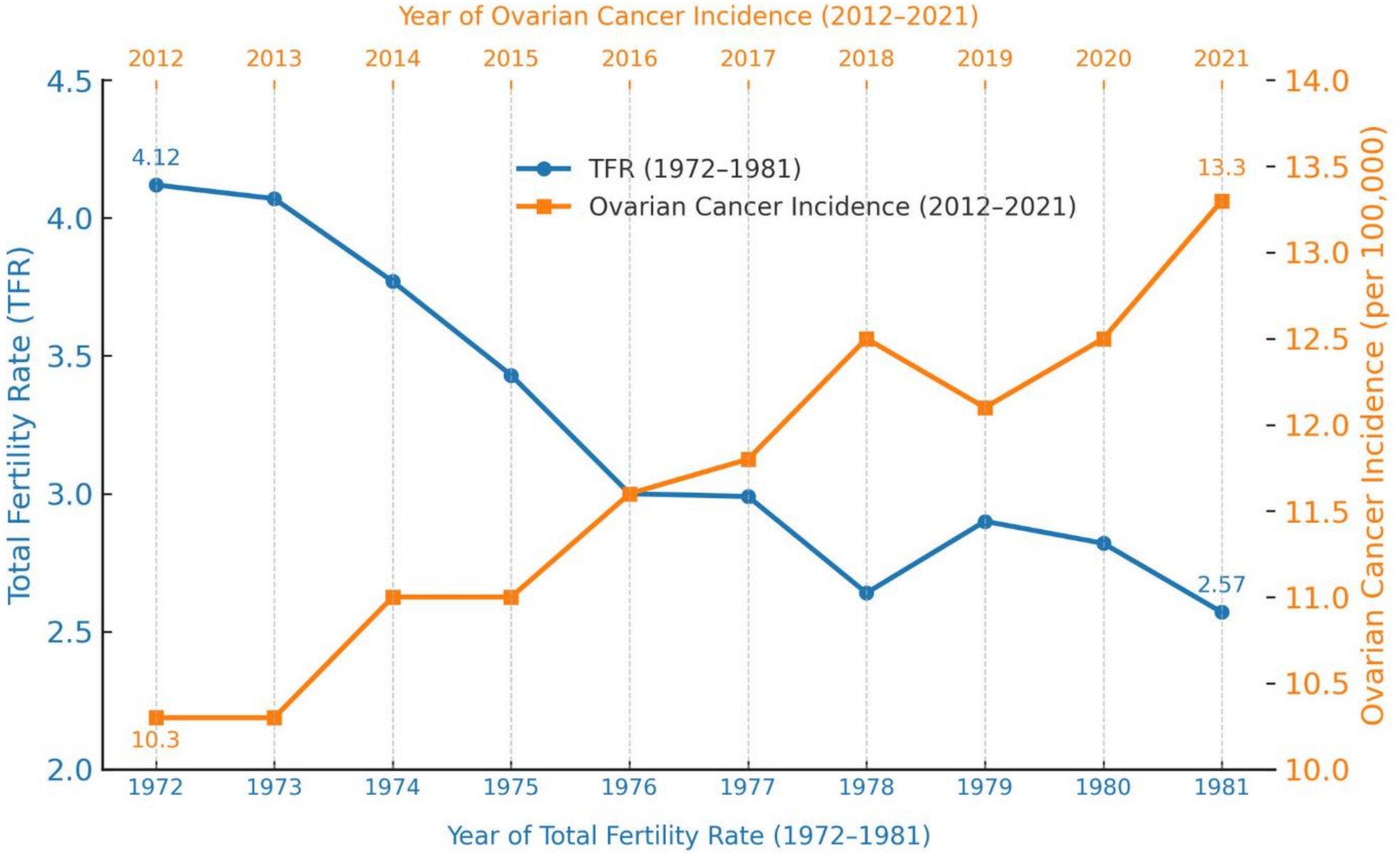

© 2026 Kim JH et al. *JAMA Network Open*
