# Supplementary material for: Reproductive Shifts and Ovarian Cancer Risk in Women Aged 40 Years or Older
Source: JAMA Netw Open. 2026 Feb 3;9(2):e2556840. doi: 10.1001/jamanetworkopen.2025.56840 (PMC12869340; doi:10.1001/jamanetworkopen.2025.56840)
Supplement: Supplement 2. — Data Sharing Statement [file jamanetwopen-e2556840-s002.pdf]

Data Sharing Statement

Kim. Reproductive Shifts and Ovarian Cancer Risk in Women Aged 40 Years or Older. *JAMA Netw Open*. Published February 03, 2026. doi:10.1001/jamanetworkopen.2025.56840

Data
Data available: No

Additional Information
Explanation for why data not available: The data that support the findings of this study are available from the Korean National Health Insurance Service but restrictions apply to the availability of these data, which were used under license for the current study, and so are not publicly available. Data are however available at https://nhiss.nhis.or.kr/bd/ay/bdaya001iv.do with the permission of Korean National Health Insurance Service.
